# Aptamers as innovative tools for malaria diagnosis and treatment: advances and future perspectives

**DOI:** 10.1093/biomethods/bpaf025

**Published:** 2025-03-27

**Authors:** Wendy Yulieth Royero-Bermeo, Miryan Margot Sánchez-Jiménez, Juan David Ospina-Villa

**Affiliations:** Instituto Colombiano de Medicina Tropical, Universidad CES, Carrera 43A 52 S-99, Sabaneta, Antioquia, 055450, Colombia; Instituto Colombiano de Medicina Tropical, Universidad CES, Carrera 43A 52 S-99, Sabaneta, Antioquia, 055450, Colombia; Instituto Colombiano de Medicina Tropical, Universidad CES, Carrera 43A 52 S-99, Sabaneta, Antioquia, 055450, Colombia

**Keywords:** malaria, aptamers, SELEX, diagnosis, treatment, HRP2, HRP3, LDH, red blood

## Abstract

Malaria, caused by *Plasmodium* spp. parasites (*P. vivax*, P*. falciparum*, *P. ovale*, *P. malariae*, and *P. knowlesi*), remains a significant global health challenge, with 263 million cases and 567 000 deaths reported in 2023. Diagnosis in endemic regions relies on clinical symptoms, microscopy, and rapid diagnostic tests. Although widely used, microscopy suffers from variability in sensitivity due to operator expertise and low parasitemia. Rapid diagnostic tests, which are favored for their simplicity and speed, show high sensitivity for *P. vivax* but reduced accuracy (80%) for *P. falciparum*, which is attributed to deletions in histidine-rich protein 2/3 proteins caused by *Pfhrp2/3* gene mutations. Innovative diagnostic and therapeutic technologies, such as aptamers, are gaining attention. Aptamers are single-stranded oligonucleotides that bind specifically to target molecules with high affinity. They have shown promise in disease diagnosis, therapeutics, and environmental monitoring. In malaria, aptamers are being explored as highly sensitive and specific diagnostic tools capable of detecting *Plasmodium* proteins across all infection stages. Additionally, they offer potential for novel therapeutic strategies, enhancing disease control and treatment options. These advancements highlight the use of aptamers as versatile and innovative approaches for addressing malaria and other infectious diseases. A comprehensive literature search was conducted in the PubMed, ScienceDirect, and SCOPUS databases via the keywords “Aptamers” AND “Malaria” AND “Aptamers” AND “Plasmodium.” Additionally, patent searches were carried out in the LENS, WIPO, and LATIPAT databases via the same search terms. In total, 88 relevant articles were selected for this review, providing a comprehensive and evidence-based foundation to discuss emerging aptamer technologies for malaria diagnosis and treatment. The proteins commonly employed in rapid malaria diagnostic tests, such as histidine-rich protein 2, *P.* lactate dehydrogenase, and prostaglandin dehydrogenase, are highlighted. However, the identification of new targets, such as HMIGB1 and DRX1 (1-deoxy-d-xylulose-5-phosphate reductoisomerase), and the detection of whole cells have also been emphasized.

## Introduction

Malaria is an acute infectious disease caused by parasites of the genus *Plasmodium*. Although numerous species within this genus have been identified, only five are known to infect humans: *P. vivax*, *P. falciparum*, *P. ovale*, *P. malariae*, and *P. knowlesi* [1]. Approximately 50% of the global population is at risk of contracting malaria. According to the World Malaria Report 2024 by the World Health Organization, 263 million cases of malaria were recorded worldwide in 2023, representing an increase of 11 million cases compared with the previous year. Of these, 567 000 cases resulted in fatal outcomes [2].

In most endemic regions of the world, where access to health care services is often limited, malaria detection is usually based on clinical symptoms, microscopic diagnosis, and immunological diagnosis through rapid tests [3]. Microscopy, although the most affordable and widely used method for malaria detection worldwide, has significant limitations. Its sensitivity depends on the analyst’s experience, which can affect accuracy in the early stages of the disease or in cases of low parasitemia [4]. These limitations have encouraged the use of rapid diagnostic tests (RDTs) for malaria to be widely utilized because of their simplicity and rapid results. Among the available options, these tests have demonstrated high sensitivity for diagnosing *P. vivax* [5]. However, recent studies assessing their performance in detecting both *P. falciparum* and *P. vivax* have shown greater accuracy in identifying the latter species [6, 7]. However, research has shown a decrease in the sensitivity of dual RDTs, reaching only 80% in the identification of *P. falciparum*. This issue has been linked to the presence of deletions in the target proteins histidine-rich protein 2 (HRP2) and histidine-rich protein 3 (HRP3) caused by the deletion of the *Pfhrp2* and *Pfhrp3* genes [8, 9]. Owing to the growing impact of this problem, government authorities have stepped up efforts to monitor it, developing strategies on the basis of data from studies focused on identifying these deletions in *P. falciparum* parasites [10].

In recent decades, biomedical research has explored a variety of innovative technologies for the diagnosis and treatment of infectious diseases, with aptamers emerging as promising alternatives [11]. Aptamers are short single-strand oligonucleotide sequences that can specifically recognize and bind to biomarkers with high affinity. These biomarkers can include cells [12], proteins [13], ions [14], and other biomolecules [15]. These molecules have demonstrated marked effectiveness in diagnosing various diseases through the precise recognition of specific pathogens [16–18]. Additionally, their versatility extends to the development of therapeutic alternatives, including the recognition and neutralization of cancer cells [19, 20], as well as their use in monitoring environmental contamination [21], standing out as an innovative technology with multiple potential applications.

**Figure 1:**
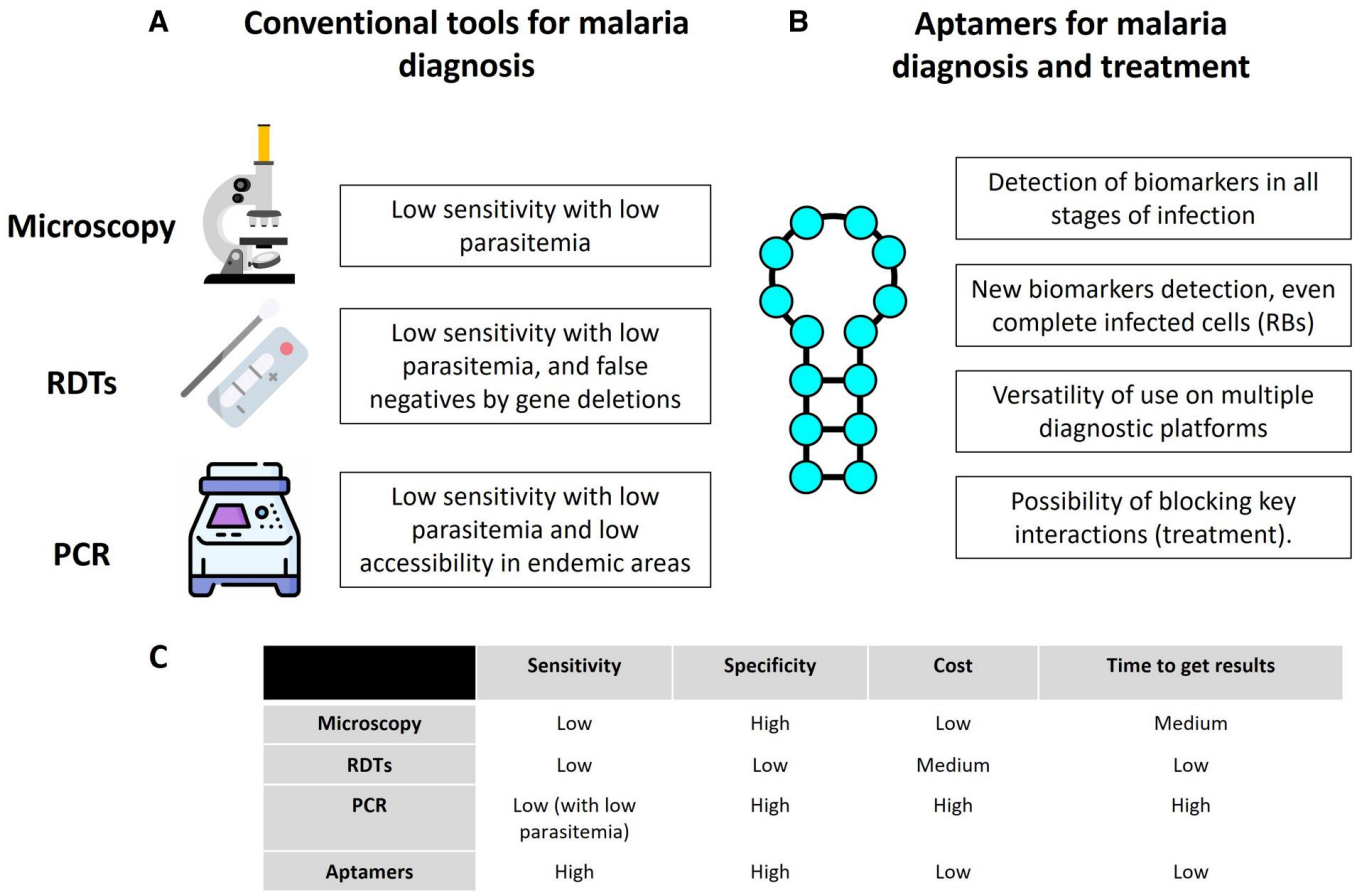
Comparison between traditional methods for malaria diagnosis and potential applications of aptamers for the diagnosis and treatment of the disease. (**A**) Conventional tools for malaria diagnosis. (**B**) Aptamers for diagnosis and treatment. (**C**) Comparison of key aspects between traditional methods and aptamers for the diagnosis of malaria

The use of aptamers in the context of malaria has focused on developing diagnostic models with higher sensitivity and specificity than currently available devices, aiming to detect *Plasmodium* spp. proteins and enable their identification at all stages of infection [22, 23]. As shown in Fig. 1, conventional diagnostic tools such as microscopy, RDTs, and polymerase chain reaction have limitations in sensitivity, specificity, or accessibility. In contrast, aptamer-based approaches offer advantages in biomarker detection, versatility, and even potential therapeutic applications, expanding possibilities for malaria diagnosis, treatment, and control.

This review delves into recent advancements in the development of diagnostic methodologies employing aptamers as molecular tools for the detection and therapeutic intervention of malaria. These innovations highlight the potential of aptamer-based approaches to increase diagnostic accuracy and contribute to the effective management of this disease.

## Materials and methods

A comprehensive literature search was conducted in the PubMed, ScienceDirect, and SCOPUS databases via the keywords “Aptamers” AND “Malaria” or “Aptamers” AND “Plasmodium.” The search was restricted to experimental studies published within the last 10 years, excluding reviews, perspectives, commentaries, and protocols. Additionally, a patent search was carried out in the LENS, WIPO, and LATIPAT databases via the same search terms without any restrictions on publication dates. Relevant data, including the type of systematic evolution of ligands by exponential enrichment (SELEX) method employed, target biomarker, dissociation constant (Kd), intended application (diagnosis, treatment, or other), *Plasmodium* species, research location, and other pertinent details, were extracted from each selected publication and patent.

## Background

The SELEX methodology, developed in 1990 by Gold, Tuerk, Ellington, and Szostak, enables the isolation of RNA molecules that specifically bind to protein targets and organic compounds [24, 25]. This technique involves generating a random nucleic acid library, followed by negative and positive selection to discard nonbinding sequences and retain high-affinity aptamers. The selected aptamers are then characterized for their affinity, stability, and interaction with biomarkers via various techniques, such as surface plasmon resonance, isothermal titration calorimetry, and fluorescence anisotropy.

SELEX has been essential for aptamer identification and design, but limitations such as multiple selection cycles have led to optimized variants [26, 27] with specific advantages such as reduced selection time, improved aptamer affinity, and enhanced specificity. Among these variants, Cell-SELEX enables aptamer selection for cell surface biomarkers [28, 29] via capillary electrophoresis to obtain high resolution and rapid separation, whereas in vivo SELEX introduces oligonucleotides into organisms to identify targets, allowing the discovery of aptamers that penetrate tissues and demonstrating its feasibility in animal models [30–32]. Capillary electrophoresis-SELEX follows the same principle as SELEX but differs in that it uses electrophoresis to separate the aptamers after incubation [33, 34]. On the other hand, the Bio SELEX methodology is based on identifying specific aptamers via biological samples [35]. The process consists of three steps: traditional SELEX, pull-down, and biomarker identification by mass spectrometry, as proposed by Ospina-Villa *et al*. [36]. Finally, the magnetic bead-based SELEX method relies on the use of magnetic beads to immobilize the target biomarker. The bound sequences are amplified to recover potential aptamers [37]. The advantages of Bio SELEX include the use of biological samples and the ability to select aptamers under physiological conditions and against native targets. Many of these variants have been widely used for the identification of aptamers against *Plasmodium* spp., standing out as highly applicable tools for the identification of potential biomarkers critical in the parasite’s life cycle, such as surface proteins, key enzymes, and other biomarkers associated with infection. This allows the identification of highly specific aptamers even under biologically relevant conditions.

The life cycle of the parasite consists of two main phases: an extraerythrocytic phase, in which the parasite takes the form of a merozoite and, through the recognition of specific proteins, invades red blood cells [38], and an intraerythrocytic phase, where it differentiates into a trophozoite and later into a schizont [39]. This last phase is particularly relevant and widely studied, as key processes such as cell division and the expression of essential proteins, including *P. falciparum* histidine-rich protein (PfHRP) and *P.* lactate dehydrogenase (pLDH), take place [40], and aptamers can be designed to target these essential proteins, disrupting the parasite’s life cycle and preventing its growth and replication. These proteins have been extensively characterized and are used as biomarkers in diagnosis and in the development of rapid tests for detecting active infections [41]. However, the use of these proteins as biomarkers has limitations, such as their potential for false-positive or false-negative results due to cross-reactivity with other proteins or genetic variations in the parasite. As the understanding of these biomarkers has advanced, strategies to treat and control malaria have increased. Currently, malaria treatment relies on the use of drugs such as chloroquine, artemisinin, and their combinations. However, increasing resistance to antimalarial drugs has created an urgent need to develop alternatives [42]. In this context, aptamers offer a promising approach to address this challenge, as they can be designed to target multiple parasite proteins or pathways simultaneously, making it more difficult for the parasite to develop resistance. In the following sections, we review the main molecular biomarkers of *Plasmodium* spp. that have been identified for aptamer development, the SELEX variants used for their characterization, and the various diagnostic formats that have been developed for aptamer-based malaria detection.

## Results

The database search yielded the following results: NCBI identified 34 articles, of which 11 were selected for their relevance, and 23 were excluded because they lacked a direct relationship with the review topic. A total of 50 articles were retrieved from PubMed, with 30 selected as relevant and 20 excluded. SCOPUS identified 57 articles, 34 of which were relevant, and 23 did not meet the selection criteria. ScienceDirect identified 79 articles, with only 13 meeting the relevance criteria, and 66 were excluded for not addressing the central topic of the review.

In total, 88 relevant articles were critically selected for this review, providing a comprehensive and evidence-based foundation to discuss emerging aptamer technologies for malaria diagnosis and treatment. The literature review revealed a growing interest in the development of aptamer-based diagnostic and therapeutic tools for malaria, with a particular focus on targeting essential parasite proteins and developing sensitive and specific diagnostic formats. Exhaustive literature analysis revealed a significant trend in the use of the SELEX methodology for the development of aptamers that target specific *Plasmodium* spp. biomarkers. The following section describes in detail the principles, fundamental stages, and applications of the SELEX process in malaria research.

### Biomarkers identified for malaria diagnosis and treatment

The identification of specific biomarkers is crucial for the development of effective aptamer-based diagnostic and therapeutic tools. These biomarkers are highly expressed by the parasite, are easily accessible for detection, and play a key role in the pathogenesis of malaria. In this section, we review several promising biomarkers that have been targeted for aptamer development.

#### HMGB1: high mobility group box 1 protein

HMGB belongs to a family of nuclear proteins that are not associated with histones, are widely conserved, and are expressed in eukaryotes. In *Plasmodium* spp., four main isoforms have been identified: HMGB1, HMGB2, HMGB3, and HMGB4 [43]. In *P. falciparum*, these proteins are highly conserved throughout all stages of the parasite [44]. Specifically, HMGB1 plays an essential role in the structural organization of the genome. Its elimination has been shown to significantly alter the architecture of key genomic regions, such as the centromere and telomere, which directly affect the expression of the *var* genes. These genes are responsible for encoding variants of *P. falciparum* erythrocyte membrane protein 1 (PfEMP1s), which play crucial roles in the pathogenesis and immune evasion of the parasite [45, 46]. For these reasons, Joseph *et al*. highlighted HMGB1 as a key factor in genome regulation and a potential therapeutic target for future interventions. In this context, they developed specific DNA aptamers through a 14-round SELEX protocol, optimizing both affinity and specificity. As a result, the Kd value of the aptamers for the HRP2 protein ranging from nanomolar to micromolar were identified: PfR6, with a Kd value of 65 ± 15 nM, showed high affinity and specificity for the HMG box of *P. falciparum*, with minimal cross-reactivity to its human homolog, and PfE3, with a Kd of 815 ± 158 nM, presented a stronger binding signal but lower affinity. Owing to the short interaction time required by the aptamer to recognize the biomarker, which was evaluated via microscale thermophoresis and stopped-flow techniques, which demonstrated rapid and efficient binding, PfR6 was selected as the optimal aptamer for the specific recognition of the HMG box of *P. falciparum*. These findings support the potential of PfR6 for integration into the development of advanced biosensors and RDTs [47].

#### HRP2

HRP2 is a protein highly expressed by *P. falciparum* during its intraerythrocytic stage. With an approximate molecular weight of 50 kDa, it is located mainly in the cytosol of infected red blood cells, where it plays a key role in the biogenesis and maintenance of parasitic vacuoles [48, 49]. This protein is secreted by the parasite during its replication cycle inside erythrocytes, occurring between 24 and 48 h after the parasite invades the erythrocyte [50]. As the parasite replicates and eventually lyses red blood cells, large amounts of HRP2 are released into the bloodstream, allowing its detection through various diagnostic tests [51]. Owing to its high abundance in the acute phase of infection, HRP2 is widely used in RDTs for malaria, providing an effective tool for the early detection of *P. falciparum* [52].

In 2018, Chakma et al. identified an aptamer named B4, characterized by its high affinity and specificity for HRP2, a crucial biomarker of *P. falciparum* that is useful for improving the sensitivity and specificity of malaria diagnosis. The aptamer was selected via a modified SELEX process, where HRP2 was immobilized on a Polyvinylidene difluoride (PVDF) membrane. After 11 rounds of positive selection and one round of counterselection, the B4 aptamer was successfully isolated. Its affinity for HRP2 was measured via isothermal titration calorimetry, which revealed a Kd of 1.32 μM. These findings highlight the potential of the B4 aptamer as a promising bioreceptor for the development of specific diagnostic tools for malaria [53]. In addition, Young Lo *et al*. expanded research on aptamers that target HRP2 by identifying a new series of DNA aptamers through a modified SELEX process, with the advantages of using DNA aptamers over RNA aptamers, such as their greater stability and lower cost of 21 selection rounds using magnetic beads functionalized with *P. falciparum* with the histidine-rich protein 2 (PfHRP2). To ensure aptamer specificity, counterselection rounds were performed against nontargeted proteins such as *P. falciparum* lactate dehydrogenase (PfLDH) and human lactate dehydrogenase isoforms (hLDHA1 and hLDHB). As a result, 37 DNA sequences capable of binding to PfHRP2 were identified. However, only one sequence, named 2106 s, was selected for the design of an electrochemical biosensor because of its outstanding affinity and specificity. The 2106 s aptamer showed a Kd of 29.53 nM in PBS buffer and 22.59 nM in diluted human serum. Its specificity has been thoroughly validated, showing minimal interaction with nontargeted proteins such as PfLDH and human serum albumin [54].

#### PfGDH: glutamate dehydrogenase of P. falciparum

The glutamate dehydrogenase (GDH) of *P. falciparum* is a key enzyme in parasite metabolism. It catalyzes the reversible oxidative deamination of L-glutamate to produce α-ketoglutarate and ammonia, using NADP(H) or NAD(H) as cofactors. Unlike human red blood cells, *P. falciparum* secretes NADPH in combination with NADP-specific isocitrate dehydrogenase, an enzyme that is not found in the host’s red blood cells. This allows the parasite to maintain its redox balance and counteract oxidative stress during its intraerythrocytic cycle [55, 56]. Scientific evidence suggests that *P. falciparum* GDH is highly conserved at the genetic level across various malaria-endemic regions [57]. Additionally, studies have shown that antibodies generated against PfGDH2 exhibit high specificity and affinity, suggesting their applicability in the design of diagnostic tests. These findings support its potential as a biomarker for the development of innovative diagnostic tools, given its evolutionary stability and specificity for the parasite [58].

Singh and colleagues identified two aptamers, NG3 and NG51, capable of specifically recognizing the PfGDH enzyme, a highly conserved protein in the sexual and asexual stages of *P. falciparum*. This makes it a promising biomarker for malaria diagnosis. They applied the SELEX technique for 17 selection rounds, including negative rounds against human glutamate dehydrogenase and positive rounds against PfGDH. As a result, two candidate aptamers were obtained: NG3, with an affinity Kd of 79.16 nM, and NG51, with a Kd of 370 nM. Finally, NG3 was selected for its high affinity and stability. The aptasensor designed with this aptamer demonstrated high specificity, with no significant interference from other malaria biomarkers, such as PfLDH and HRP2. It achieved a detection limit (LOD) of 0.77 pM in human serum. This progress highlights the potential of PfGDH as a diagnostic biomarker and the viability of aptamers as robust and precise tools in low-resource settings [59].

### PfDXR: 1-deoxy-d-xylulose-5-phosphate reductoisomerase of P. falciparum

The 1-deoxy-d-xylulose 5-phosphate reductoisomerase of *P. falciparum* (*PfDXR)* is a homodimeric enzyme located in the apicoplast of the parasite [60], also known as IspC, which is derived from the *ispC* gene in the isoprenoid biosynthesis pathway. This enzyme plays a crucial role in the conversion of the substrate 1-deoxy-d-xylulose 5-phosphate into 2-C-methyl-d-erythritol 4-phosphate (MEP), a key intermediate in the nonmevalonate (MEP) pathway [61]. Several studies have shown that this enzyme is essential for the intraerythrocytic development of *P. falciparum* [62], and drugs such as fosfomycin, which is currently used in malaria treatment, can inhibit its activity [63, 64].

In the work by Roca et al., the aptamer D10, identified via the Mag-SELEX strategy, shows high specificity for the enzyme DXR, a key component of the MEP pathway, which is essential for the synthesis of isoprenoids in the parasite’s apicoplast. With a Kd of 260 nM, D10 binds efficiently to DXR, although its inhibitory capacity is limited (IC50 = 9.6 μM) because it primarily interacts with exposed regions of the enzyme without completely blocking the active site. The D10 aptamer offers promising potential in diagnostics, as it specifically discriminates parasite-infected red blood cells from uninfected red blood cells. This level of specificity opens the door to its integration into advanced diagnostic platforms for malaria detection. Additionally, the authors suggested that D10 could be chemically modified by incorporating inhibitory molecules, such as fosmidomycin analogs, to improve its therapeutic action [65].

### Var2CSA: specific variant of the PfEMP1 family

PfEMP1 is encoded by the var (∼60 genes), rif (∼150 genes), and stevor (∼30 genes) gene families [66], which play crucial roles in the adhesion of the parasite to specific endothelial membrane proteins, such as ICAM-1, CD36, and endothelial protein C receptor (EPCR), facilitating its sequestration in the microvasculature and rosette formation and contributing to the severe pathogenesis of *P. falciparum* malaria [66–68]. Furthermore, their involvement in cerebral malaria processes has been demonstrated through increased expression of the EPCR and its interaction with PfEMP1 [69]. The antigenic variability of PfEMP1, resulting from the alternative expression of var genes, enables *P. falciparum* to evade the host immune response and prolong infection by continuously modifying protein epitopes [70].

Given the critical role of PfEMP1 in malaria pathogenesis and immune evasion, efforts have been made to identify specific biomarkers that can serve as diagnostic and therapeutic biomarkers. In this context, Birch et al. (2015) employed the I-SELEX technique, which is based on inertial microfluidics, to select aptamers that target *P*. *falciparum*-infected red blood cells (pRBCs). This method led to the identification of aptamer 8.1–1, which demonstrated high affinity for the biomarker Var2CSA, with a Kd of 14 nM. Var2CSA, a specific variant of the PfEMP1 protein family, is expressed on the surface of infected erythrocytes and plays a critical role in the pathogenesis of severe malaria. The authors highlight the potential of these aptamers not only as diagnostic tools for detecting specific markers on infected erythrocytes but also as therapeutic alternatives. In this context, aptamers can block key interactions between infected erythrocytes and host cells, reducing severe complications such as placental malaria. This approach complements previous advancements in aptamer development, underscoring their relevance in the search for innovative strategies for malaria diagnosis and treatment [23].

### pRBCs: *Plasmodium* spp. infected red blood cells

During the intraerythrocytic phase of *Plasmodium* spp., red blood cells undergo structural changes that induce the expression of proteins on their membrane, such as *mature parasite-infected erythrocyte surface antigen*, which act as antigens [71]. Additionally, the presence of PfEMP3 contributes to increased cell rigidity [72]. These structural alterations cause cell deformation and affect circulation dynamics, which may compromise blood flow and tissue [73]. They also facilitate the adhesion of infected cells to the vascular endothelium, a key process in severe malaria pathogenesis mediated by the expression of the *PfEMP1* protein [74]. These modifications in the erythrocyte membrane make these proteins potential biomarkers for the development of aptamers capable of recognizing the entire cell or specific proteins.

Owing to this relevance, numerous studies have explored the application of the Cell-SELEX methodology for the identification of specific aptamers. A notable example is the work of Lantero et al. (2020), who used the Cell-SELEX technique to develop highly specific aptamers that target pRBCs. After 10 selection cycles, they identified five main aptamers (19, 24, 30, 77, and 78), which presented binding rates above 84.5% and minimal nonspecific binding (≤0.06%). These aptamers exhibited high affinity for uncharacterized intracellular biomarkers associated with the late stages of the intraerythrocytic cycle. The Kd values ranged from 0.46 to 1.77 µM, indicating strong interactions with their targets. Although initially designed for *P. falciparum*, they also recognized all developmental stages of *P. vivax*, *P. ovale*, and *P. malariae*, highlighting their potential for the development of broad-spectrum diagnostic tools with high specificity and clinical relevance [71].

### pLDH: lactate dehydrogenase of *Plasmodium* spp.

LDH is an essential enzyme in cellular metabolism that catalyzes the conversion of pyruvate to lactate and the simultaneous interconversion of NADH to NAD+. In the context of malaria, this enzyme is highly expressed, reaching its maximum accumulation inside erythrocytes between 10 and 20 hours after infection, specifically during the late ring stage [40]. Compared with its human counterpart, the LDH of *Plasmodium* spp. shows structural, biochemical, and functional differences, as well as variability between species. It has been reported that the pLDHs of *P. vivax, P. malariae, and P. ovale share 90%–92% genetic identity with the pLDH of P. falciparum, which facilitates the differentiation of these species in diagnostic tests* [75, 76]. Owing to its high concentration in infected individuals and its remarkable genetic conservation, LDH is one of the most widely used biomarkers [77, 78]. Over the years, various diagnostic formats using pLDH to detect the presence of *Plasmodium* in biological samples have been developed, enabling the implementation of accessible and effective diagnostic methods [75, 79, 80].

For these reasons, LDH has been one of the most studied and utilized enzymes in the development of aptamers, making it a key target for research into more precise and rapid diagnostics. Several studies have identified specific aptamers that target the pLDH of *Plasmodium*, which has opened new possibilities for the early detection of this disease. The following section describes the most relevant advancements in this field, including the aptamers discovered and their potential applications.

In 2012, Lee et al. used the SELEX method with magnetic beads to immobilize the lactate dehydrogenase protein of *P. vivax* (PvLDH). This method helps remove nonspecific oligonucleotide sequences in each selection cycle by using an external magnet. As a result, they identified two promising aptamers able to recognize PvLDH. The first aptamer, called PL1, had a Kd of 16.8 nM for PvLDH and 38.7 nM for PfLDH. The second aptamer, named PL2, had Kd values of 31.7 nM for PvLDH and 49.9 nM for PfLDH. These results show the high affinity and specificity of the aptamers PL1 and PL2 for LDH proteins from *P. vivax* and *P. falciparum*. This makes them promising tools for the development of new methods to detect malaria accurately [76]. Subsequently, by affinity chromatography, Cheung et al. (2013) selected aptamers to target PfLDH immobilized on magnetic beads. Cheung et al. (2013) subsequently used the SELEX methodology, which incorporates counterselection against human LDH, to isolate and characterize the DNA aptamer called 2008s, with a Kd of 42 nM for PfLDH. This aptamer showed high specificity for PfLDH, making it a promising tool for malaria diagnosis. They also tested its diagnostic applicability via a colorimetric detection system based on conjugation with gold nanoparticles, demonstrating effective discrimination between PfLDH and human LDH. These results highlight its potential as a foundation for developing aptamer-based diagnostic tests for accurate malaria detection [81].

Jain et al. (2016) reported the identification of a ssDNA aptamer named P38, developed through the traditional SELEX methodology, that incorporates counterselection steps to avoid nonspecific interactions with hLDH isoforms A and B. This aptamer showed specific binding ability to PfLDH, with a Kd of 0.35 μM, highlighting its potential for diagnostic applications specific to *P. falciparum* [82]. Subsequently, Godonoga *et al*. identified specific aptamers against the PfLDH protein via SELEX methodology. These aptamers were modified via the DNA origami scaffold technique introduced by Paul Rothemund in 2006, which enables the generation of highly precise and programmable three-dimensional structures from DNA molecules. The modified aptamers exhibited affinity for the protein, with Kd values ranging from 647 ± 128 nM to 1090 ± 183 nM. Their integration into DNA origami structures was explored, highlighting their potential as advanced diagnostic devices for malaria. Notably, the incorporation of aptamers into these platforms opens new possibilities for the development of biosensors capable of specifically detecting PfLDH and even evolving into more complex systems that release detectable signals in the presence of the biomarker [22, 83].

Frith *et al*. focused on developing highly specific PfLDH aptamers via a modified SELEX technique based on nitrocellulose. Through this process, they identified two sets of aptamers targeted against the recombinant PfLDH protein (rPfLDH). The first set, called recombinant LDH 4 (rLDH 4), had a Kd of 691.6 nM for rPfLDH and 444.9 nM for recombinant PvLDH (rPvLDH). The second set, rLDH 15, had a Kd of 80.7 nM for rPfLDH and 268.7 nM for rPvLDH. In addition, they identified aptamers targeting the LDH epitopic oligopeptide (LDHp) peptide, with LDHp 11 showing a Kd of 321.2 nM for rPfLDH and no significant binding to rPvLDH. This aptamer, LDHp 11, is a promising candidate for use in diagnostic devices because of its high specificity for rPfLDH and lack of cross-reactivity with rPvLDH. In comparison, the rLDH 4 aptamer showed good affinity for rPfLDH but also had some binding to rPvLDH, suggesting lower specificity than LDHp 11 [84]. Cheung *et al*. reported the development of a “cubamer,” an aptamer modified with cubane, a cyclic compound containing carbon atoms that form part of its ring structure. This cubamer was specifically directed at PvLDH, a crucial biomarker for malaria diagnosis. The aptamer exhibited an affinity of 670 ± 9 nM for PvLDH and high selectivity, demonstrating a 30-fold lower affinity for PfLDH, despite the considerable homology between the two proteins. This finding highlights the potential of the cubamer to distinguish between *Plasmodium* species, which is essential for improving diagnostic accuracy [85]. However, Chung *et al*. developed an innovative method for aptamer selection using an electrodynamic microfluidic channel. In this system, the PvLDH protein was immobilized on the walls of the device while a single-stranded DNA (ssDNA) library was introduced, and electrodynamic forces were applied to increase collisions between ssDNA molecules and PvLDH. This process favors the specific binding of potential aptamers to biomarkers. The bound sequences were subsequently eluted, identifying five aptamers, L1 (Kd: 31.4 nM), L2 (Kd: 97.3 nM), L3 (Kd: 82.6 nM), L4 (Kd: 78.5 nM), and L5 (Kd: 43.5 nM), with aptamer L1 standing out for its high affinity toward the biomarker. This innovative approach is proposed to significantly accelerate the aptamer selection process, completing it in a single three-hour cycle, in contrast to traditional methods that require multiple rounds [86].

Finally, in 2021, Kantor *et al*. presented an innovative method for capturing and purifying *P. falciparum*-specific biomarkers, utilizing high-affinity aptamers, specifically pL1 and 2008s, coupled to magnetic beads for the identification and concentration of the PfLDH and PvLDH proteins, respectively. The biomarker-enriched aptamers pL1 and 2008s were directly applied to commercial RDTs for malaria, enabling the evaluation of improvements in diagnostic sensitivity. The results demonstrated a significant increase in the detection of PfLDH, with a LOD of 3.8 parasites/µL, which represents an 11-fold increase in sensitivity compared with the 41.7 parasites/µL detected by RDTs without enrichment. This breakthrough highlights the AnDREW protocol as a promising tool for early and sensitive malaria diagnosis, particularly in resource-limited settings [87].

## Diagnostic formats for *Plasmodium* spp. detection

**Figure 2:**
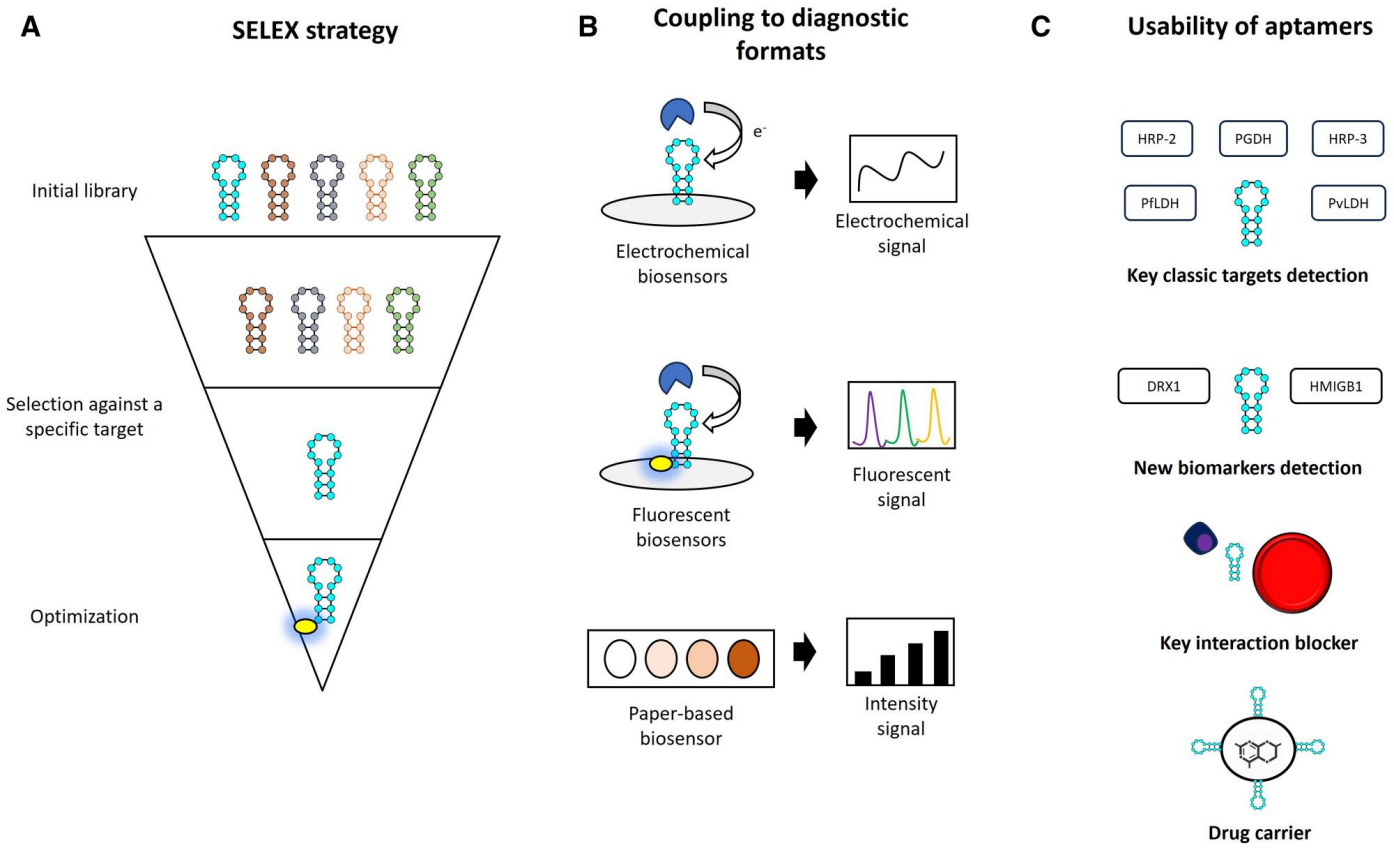
Overview of aptamers in the diagnosis and treatment of malaria. (**A**) Summary of the SELEX strategy for the development of aptamers. (**B**) Adaptation of the aptamers in different diagnostic formats. (**C**) Potential use of the aptamers for malaria control

In recent years, various aptamer-based diagnostic devices, such as PLDH, PfGDH, and PfHRP2, have been developed for the detection of key *Plasmodium* spp. biomarkers. Among the most prominent formats are electrochemical biosensors, microfluidic devices, and colorimetric assays, which are characterized by high sensitivity, with LODs ranging from femtomolar (fM) to nanomolar (nM) levels in both human samples and laboratory simulations. Additionally, these devices offer significant advantages for portable applications, making them particularly suitable for resource-limited settings (Fig. 2).

Despite these advancements, several challenges remain, including variability in clinical samples and the need for large-scale clinical validation to ensure their implementation in real-world contexts. Nevertheless, these technologies represent a significant step forward in the early and accurate diagnosis of malaria, with great potential for integration into public health programs.

The following section provides a comprehensive review of diagnostic devices developed from 2014 to the present (Table 1).

**Table 1.** Summary of malaria diagnostic formats based on aptamers reported in the literature.

Biomarker	Device	Sensitivity	Observation	Reference
**PLDH**	Microfluidic paper analytical device (µPAD)	17 nM of rPfLDH 133 nM	Recombinant protein Serum undiluted	[80]
	Gold nanoparticle aggregation-based colorimetric assay	281 ± 11 pM	Benzalkonium chloride	[82]
	Fluorescent biosensor based on gold bimetallic nanoclusters	0.10 nM (3.7 ng/mL)	Protein diluted in fetal bovine serum	[88]
	Electrochemical impedance spectroscopy	(1 pM–100 nM)	rPvLDH and buffer	[89]
	Surface plasmon polariton	10 nM–1 μM		
	Aptasensor based on plasmon-enhanced fluorescence	0.3 ng/mL–300 μg/mL	Total blood	[90]
	Aptasensor FRET based on MoS2 nanosheets to detect *Plasmodium* spp. lactate dehydrogenase.	19.2 ng/mL (550 pM)	Better than antibody-based methods (25–50 ng/mL)	[91]
	Colorimetric GNP–aptamer assay	1.25 pM for *Pv*LDH 2.94 pM for *Pf*LDH	Serum	[92]
	Ultrasensitive aptamer-antibody plasmonic biosensor	1 pg/mL (<30 fM)	Total blood	[93]
	Laboratory format: spectrophotometry and spectrofluorimetric	0.41 ± 0.07 pM absorption 1.63 ± 0.14 pM fluorescence	Serum	[94]
	Portable format without instruments. Colorimetric	61.50 ± 6.43 pM 69.25 ± 8.22 pM	Buffer Serum	
	Aptamer-based electrochemical biosensor for highly sensitive and selective malaria	1.30 pM	Diluted serum	[95]
	Microfluidic portable aptamer-bound enzyme capture (APTEC) biosensor	250 parasites/µL	Total blood	[96]
	Aptasensor based on reduced graphene field effect transistors (2DBioFETs)	0.78 fM (0.11 pg/mL)	Serum	[97]
	Polyethylene glycol-mediated blockade and monolayer morphology of an electrochemical aptasensor	1.49 pM.	Serum	[98]
	APTEC: aptamer-tethered enzyme captures as a novel RDT for malaria	4.9 ng mL	Total blood	[99]
	Aptasensor based on graphene oxide	0.5 Fm/105 red blood infected	Superior sensitivity to colorimetry/whole blood-based methods	[100]
	Apta-chip-basd immunoassay	50 pM (1,6 ng/mL) with 5-FAM/260 fM (8,6 pg./mL) with Cy5	Total blood	[101]
	Electrochemical aptasensor	7.8 ± 0.4 fM	Recombinant protein	[86]
	Scaffolded silver nanoclusters combined with an aptamer	7.4 pg/μL	Recombinant protein	[102]
	Aptamer-based sandwich assay for the detection of lactate dehydrogenase	0.5 fM in buffer	–	[103]
	Gold nanoparticles functionalized with oligonucleotide	10 nM		
**PfGDH**	Cubamers	0.77 pM	Serum	[59]
	Capacitive sensor suitable for *P. falciparum Diagnosis*	> 1 nM		
	Smartphone-based, multichannel, optic fiber platform for quantitative detection of *P. falciparum* glutamate dehydrogenase	264 pM	Serum	[104]
	Detection of *P. falciparum* glutamate dehydrogenase using carbon dot coupled specific aptamer.	2.85 nM	Diluted serum	[105]
	Aptamer-based field effect transistor biosensor	16.7 pM 48.6 pM	Buffer Serum	[106]
	Laboratory format spectrophotometry and spectrofluorimetry	0.81 pM fluorescence 1.14 ± 0.12 pM absorption	Serum	[94]
	Portable format without instruments. Colorimetric	63.97 ± 7.24 pM 68.75 ± 7.64 pM	Buffer Serum	
**PfHRP2**	An electrochemical aptamer-based biosensor targeting *P. falciparum* histidine-rich protein 2	3,73 Nm	Serum	[54]
	Electrochemical impedance spectroscopy based malaria aptasensor using HRP2 as target biomarker	3.15 Pm	Diluted serum	[53]
**Multiplex**	flex-MEA multi-target aptasensor	1.80 Fm—PfLDH 0.5 Pm—HRP2	Multi-target 4 aptamers	[107]

## Patents

In recent years, the development of specific aptamers for the detection of *Plasmodium* spp. biomarkers has generated significant interest, as reflected in many patent applications worldwide. These patents focus on diagnostic and therapeutic innovations for malaria via aptamers, employing various SELEX methodology variants, with traditional SELEX being the most common, followed by Cell-SELEX and magnetic particle-based SELEX. The applications come from countries in America, Europe, and Asia, covering key biomarkers such as HMGB1, PLDH, PfLDH, and HRP2, demonstrating a global effort to combat malaria through novel technologies.

The following section summarizes some of the most relevant patents for the detection of *Plasmodium* spp. biomarkers (Table 2).

**Table 2.** Summary of the most relevant published patents using aptamers for the diagnosis and treatment of malaria.

SELEX	Biomarker	Utility	Species	Country	Reference	Database
Cell-SELEX	*pRBC—*red blood cells infected	Diagnosis	*Plasmodium* spp.	Spain	WO/2021/180906	LENS
Traditional SELEX	HMGB1	Diagnosis/treatment	*P. falciparum*	Peru	PE20191051	LATIPAD
Traditional SELEX	EP1 PROTEIN	Diagnosis/treatment	*P. falciparum*	Peru	PE20191052	LATIPAD
Traditional SELEX	TS1 PROTEIN	Diagnosis/treatment	*P. falciparum*	Peru	PE20191053	LATIPAD
Traditional SELEX	CPP1 PROTEIN	Diagnosis/treatment	*P. falciparum*	Peru	PE20190572	LATIPAD
Traditional SELEX	PLDH	Diagnosis/treatment	*P. falciparum/vivax*	Hong Kong	WO/2019/113827	LENS- WIPO
Magnetic beads-based SELEX	PfLDH	Diagnosis	*Plasmodium* spp.	Korea	EP2532749	LENS-WIPO
Traditional SELEX	PLDH	Diagnosis	*Plasmodium* spp. *P. vivax*	USA	US20120316325	WIPO
Traditional SELEX	PLDH	Diagnosis	*Plasmodium* spp.	China	CN102816763	WIPO
Traditional SELEX	PLDH	Diagnosis	*Plasmodium* spp.	Japan	JP2012254074	WIPO
Traditional SELEX	PfGDHA	Diagnosis	*P. falciparum*	India	IN201631025722	WIPO
Traditional SELEX	PfEM1	Diagnosis/treatment	*P. falciparum*	Sweden	EP2247728, WO2009099378, US20160040164, ES2537201, US20160032293, US20110003886	LENS—WIPO
Magnetic beads-based SELEX	HRP2/PLDH	Diagnosis	*P. falciparum Plasmodium* spp.	Hong Kong	US9000137	LENS

The growing use of aptamers in biomedicine, biotechnology, and pharmaceutical development has greatly boosted research and commercialization of aptamer-based technologies. This increase is shown by a steady rise in patents and innovative products worldwide, with strong interest in developing biosensors, targeted therapies, and molecular detection platforms. As a result, the global aptamer market has grown rapidly, reaching a value of USD 1.94 billion in 2022 and is expected to grow at a compound annual rate of 24.54% from 2023 to 2030 [108]. Key players in this market include companies such as SomaLogic, Aptamer Group, Aptadel Therapeutics, Base Pair Biotechnologies, Noxxon Pharma, Vivonics Inc., and Aptagen, LLC. These organizations are at the forefront of developing and commercializing aptamer-based diagnostics and therapies, and they significantly contribute to patent filings and technological advancements in the field [109].

## Conclusions and future perspectives

Aptamers represent an innovative alternative in the development of diagnostic tests because of their high sensitivity and ability to recognize a wide variety of targets, regardless of their biological nature. Current evidence suggests that these methods can overcome some of the limitations of conventional techniques, especially in terms of specificity and LODs. For instance, while microscopy has a as a sensitivity of 65.3% and specificity 98.2%, under optimal laboratory conditions, aptamer-based methods have shown the potential for even lower LODs [110]. One example of this is the use of MoS_2_ nanosheets in FRET assays, which have shown higher sensitivity than traditional immunoassays, with a LOD of 19.2 ng/mL compared with 25–50 ng/mL in conventional methods [91].

In the context of malaria diagnosis, the sensitivity of commercial tests varies significantly depending on the format used and the biomarker detected. Throughout this review, the use of proteins commonly employed in RDTs for malaria, such as HRP2, PLDH, and prostaglandin dehydrogenase, has been highlighted. However, emphasis has also been placed on identifying new biomarkers, such as HMGB1 and DRX1, as well as detecting whole cells. Nevertheless, there is still a need to develop large-scale assays and identify new biomarkers capable of recognizing the parasite in each of its stages, which remains a key area of research. A major limitation in malaria diagnostics is the deletion of the *PfHRP2* gene, which impacts the performance of HRP2-targeting assays, including both traditional RDTs and aptamer-based approaches. However, aptamers exhibit a unique advantage due to their high specificity and strong affinity for target molecules, relying on structural conformational changes for enhanced detection accuracy. To overcome the challenge posed by *PfHRP2* deletions, most recent studies have focused on alternative biomarkers, such as *Plasmodium falciparum* lactate dehydrogenase (pLDH), and novel targets like HMGB1 and DRX1, which remain unaffected by these deletions. Nevertheless, research on aptamers specifically designed for HRP2 detection is still limited.

Although large-scale studies on the sensitivity and specificity of aptamer-based sensors are still lacking, available data suggest they have a superior LOD compared to traditional methods. A FRET-based aptasensor has detected pLDH at 550 pM, outperforming antibody-based methods (25–50 ng/mL), which show reduced sensitivity at parasitemias below 50 parasites/µL [91]. Additionally, aptamers have shown better detection in low parasitemias, identifying pLDH at 3.80 parasites/µL, 11 times more sensitive than conventional RDTs and HRP2 with a 9-fold improvement compared to the 42.31 parasites/µL detected by the evaluated RDT [87]. These findings highlight their potential to improve malaria diagnosis, especially in cases of low parasite load.

While antibodies have been widely used in diagnosis, their production is a complex and costly process compared with aptamer synthesis. Additionally, antibodies are more prone to denaturation due to their protein structure. In contrast, aptamers offer significant advantages, such as shorter development times, lower batch-to-batch variability, and greater stability, which strengthens their potential as innovative tools for diagnosing infectious diseases. Owing to their ability to recognize almost any type of biomolecule, aptamers can be designed using novel biomarkers of various natures, expanding their applications in the development of diagnostic technologies. Importantly, numerous factors present in the sample, or the patient, can affect the sensitivity and specificity of tests. Factors such as a low parasitic load that exceeds the minimum LOD of the test or a high parasitic load that causes a prozone effect may lead to false-negative results. Additionally, geographic variability in *Plasmodium* spp. strains, specific mutations in target biomarkers, and the presence of underlying conditions that alter the composition of the sample with interfering metabolites present significant challenges. Therefore, it is essential to conduct robust clinical trials that validate the performance of aptamer-based diagnostic formats in real-world conditions, ensuring their applicability in different epidemiological settings. This is just one of the many challenges that this emerging technology faces.

Optimizing the stability and functionality of aptamers in tropical environments is critical for their implementation in rapid malaria diagnosis. Furthermore, the development of aptamers capable of detecting *Plasmodium* spp. in all stages, both sexual and asexual, would broaden their applicability in the comprehensive diagnosis of this disease.

Despite their advantages, using aptamers for malaria diagnosis faces key challenges. One of the main issues is cost. While aptamers can be more affordable than monoclonal antibodies in the long run, their initial research and production costs are high, which may limit their use in low-resource settings. Another challenge is stability [111]. Aptamers can be degraded by nucleases, affecting their performance in biological samples. To solve this, chemical modifications and proper storage conditions are necessary [112]. Lastly, large-scale implementation requires strict validation, regulatory approval, and integration into existing diagnostic systems, which can be complex and costly. Overcoming these challenges is essential for making aptamers a reliable and effective tool for malaria diagnosis, especially in endemic areas.

### Potential therapeutic applications of aptamers in malaria

Given the diversity of target proteins recognized by aptamers and their involvement in key biological processes, their potential therapeutic application in malaria is promising. Below are some of their possible applications:

#### Inhibition of cytoadhesion

Aptamers targeting the membrane protein of *P. falciparum*-infected erythrocytes (PfEMP1) could block the adhesion of parasitized red blood cells to the vascular endothelium. This would reduce microvascular obstruction and mitigate severe complications associated with malaria, such as cerebral malaria [113].

#### Blocking essential metabolic pathways

Aptamers designed to recognize PfLDH and DXR could interfere with critical metabolic processes for parasite survival. Inhibiting PfLDH would affect energy production, while blocking DXR would interrupt the synthesis of isoprenoids, essential for various cellular functions [114].

#### Adjunct therapy

Beyond their antiparasitic effect or potential diagnostic use, aptamers can act as adjunct therapies, enhancing the efficacy of existing antimalarial drugs and helping to counteract drug resistance. Their ability to modulate the host-pathogen interaction allows for the exploration of combined therapeutic strategies with better clinical outcomes [115].

One of the main future perspectives for the use of aptamers in malaria diagnosis is the design of detection platforms aimed at strains with deletions in the *pfhrp2/pfhrp3* genes. The absence of these biomarkers compromises the sensitivity of conventional diagnostic tests, so these tools would improve diagnostic accuracy and facilitate epidemiological monitoring and geographic surveillance of circulating parasite variants. Finally, another emerging application of aptamers is their ability to detect mutations associated with resistance to antimalarial drugs. This capability would allow for the development of rapid and accessible tests to assess parasite susceptibility to available treatments, facilitating more efficient decision-making in disease management.

## Author contributions

Wendy Yulieth Royero-Bermeo (Conceptualization [equal], Data curation [equal], Formal analysis [equal], Investigation [equal], Writing—original draft [equal]), Miryan Margot Sánchez-Jiménez (Conceptualization [supporting], Supervision [equal], Writing—review & editing [equal]), and Juan David Ospina-Villa (Conceptualization [equal], Formal analysis [equal], Funding acquisition [equal], Investigation [equal], Project administration [equal], Resources [equal], Supervision [equal], Validation [equal], Writing—original draft [equal], Writing—review & editing [equal])

*Conflict of interest statement*. The authors declare that they have no competing interests.

## Funding

This work was supported by CES University, Internal Call 2024-INV.042024.007.

## Data Availability

No new data were generated or analysed in support of this research.
